# Store-Operated Ca^2+^ Entry Plays a Role in HMGB1-Induced Vascular Endothelial Cell Hyperpermeability

**DOI:** 10.1371/journal.pone.0123432

**Published:** 2015-04-17

**Authors:** Mengchen Zou, Hangming Dong, Xiaojing Meng, Chunqing Cai, Chenzhong Li, Shaoxi Cai, Yaoming Xue

**Affiliations:** 1 Department of Endocrinology and Metabolism, Nanfang Hospital, Southern Medical University, Guangzhou, 510515, China; 2 Department of Respiratory and Critical Care Medicine, Chronic Airways Diseases Laboratory, Nanfang Hospital, Southern Medical University, Guangzhou, 510515, China; 3 Department of Occupational Health and Occupational Medicine, School of Public Health and Tropical Medicine, Southern Medical University, Guangzhou, 510515, China; Penn State Hershey College of Medicine, UNITED STATES

## Abstract

**Aims:**

Endothelial dysfunction, including increased endothelial permeability, is considered an early marker for atherosclerosis. High-mobility group box 1 protein (HMGB1) and extracellular Ca2+ entry, primarily mediated through store-operated Ca2+ entry (SOCE), are known to be involved in increasing endothelial permeability. The aim of this study was to clarify how HMGB1 could lead to endothelia hyperpermeability.

**Methods and Results:**

We have shown that human vascular endothelial cell permeability is increased, while transendothelial electrical resistance and VE-cadherin expression were reduced by HMGB1 treatment. Two SOCE inhibitors and knockdown of stromal interaction molecule 1 (STIM1), a Ca^2+^ sensor mediating SOCE, inhibited the HMGB1-induced influx of Ca^2+^ and Src activation followed by significant suppression of endothelial permeability. Moreover, knockdown of Orai1, an essential pore-subunit of SOCE channels, decreased HMGB1-induced endothelial hyperpermeability.

**Conclusions:**

These data suggest that SOCE, acting via STIM1, might be the predominant mechanism of Ca^2+^ entry in the modulation of endothelial cell permeability. STIM1 may thus represent a possible new therapeutic target against atherosclerosis.

## Introduction

Atherosclerosis remains one of the most important and common causes of death and disability in developed countries [1, 2]. It has been predicted that atherosclerosis will be the main cause of mortality and disability in the world by 2020 [3]. Atherosclerosis is an inflammatory condition characterized by progressive thickening of the arterial wall due to the accumulation of lipids [4]. An initial phase of the atherosclerotic process involves endothelial dysfunction, with subsequent increases in endothelial permeability [4–6]. High-mobility group box 1 protein (HMGB1) has been reported to act as a pro-inflammatory factor mediating chronic inflammatory responses in endothelial cells, which in turn play a critical role in atherosclerosis[7–9]. Circulating HMGB1 concentrations are elevated in patients with atherosclerotic coronary artery diseases [10–12]. Evidence has shown that HMGB1 increases the hyperpermeability of endothelial cells in sepsis and acute lung inflammation [13, 14]. However, the precise mechanisms by which HMGB1 regulates endothelial hyperpermeability in atherosclerosis remain to be established.

Intracellular Ca^2+^ plays a critical role in endothelial permeability[15], regulated in part by the coordinated opening and closing of cell-cell adhesion junctions composed largely of vascular endothelial (VE)-cadherin [16]. VE-cadherin is an endothelium-specific member of the cadherin family and a Ca^2+^-dependent cell adhesion molecule expressed in atherosclerotic lesions [17]. Levels of Ca^2+^ signaling in endothelial permeability are regulated by a mechanism known as store-operated Ca^2+^ entry (SOCE) [18], which represents a major Ca^2+^ influx pathway in most non-excitable cells [19]. SOCE is activated by depletion of Ca^2+^ stores in the endoplasmic reticulum (ER) and is mediated essentially by two classes of proteins, stromal interaction molecule (STIM) and Orai proteins [20, 21]. Previous studies have shown that SOCE activation was required to increase permeability in pulmonary artery endothelial cells [22, 23]. Nonetheless, it remains unknown if SOCE exerts control over endothelial hyperpermeability regulated by HMGB1 in atherosclerosis.

Several reports have shown that Src family kinases play a role in thapsigargin (TG)-evoked SOCE [24–26] and are involved in HMGB1-induced hyperpermeability [13]. In the current study, we investigated the ability of HMGB1 to increase the permeability of human vascular endothelial cells (EA.hy926). To determine the role of SOCE in HMGB-1 induced endothelial hyperpermeability, we used the well known SOCE inhibitors [27, 28], SKF96365 and 2-aminoethoxydiphenyl borate (2-APB) which blocks Ca^2+^ entry and IP3 receptor respectively. We also knocked down STIM1 expression by small interfering RNA (siRNA) to investigate the role of SOCE in this process, and examined the abilities of both SOCE inhibitors and STIM1 knock-down to affect Ca^2+^ influx and Src activation. The results of this study clarify the role of SOCE in the regulation of Src kinase activity during vascular permeability.

## Material and Methods

### Reagents and antibodies

HMGB1, PP2, CGP77675, SKF96365, 2-APB, thapsigargin (TG) and dimethyl sulfoxide (DMSO) were purchased from Sigma Chemical Co. (St. Louis, MO, USA). Fluo-4/AM and CCK-8 Kits were purchased from Dojindo Laboratories (Kumamoto, Japan). Dulbecco's modified Eagle's medium (DMEM) and fetal bovine serum (FBS) were from Gibco (Grand Island, NY, USA). Lipofectamine 2000 was from Invitrogen (Carlsbad, CA, USA). FITC-labeled secondary antibodies, mouse monoclonal anti-glyceraldehyde 3-phosphate dehydrogenase (GAPDH) antibody and 4',6-diamidino-2-phenylindole (DAPI) were from Santa Cruz Biotechnology (CA, USA). Rabbit polyclonal anti-VE-cadherin antibody, rabbit polyclonal anti-Na,K-ATPase α1 antibody, horseradish peroxidase (HRP)-conjugated goat anti-rabbit antibody, rabbit polyclonal anti-phospho-Src antibody and rabbit polyclonal anti-Src antibody were from Cell Signaling Technology (Beverly, MA, USA). Rabbit polyclonal anti-STIM1 antibody, and anti-human Orai1 antibody were from Abcam (Cambridge, MA, USA).

### Cell cultures

The human umbilical vein endothelial cell line EA.hy926 (provided by China Center for Type Culture Collection, Shanghai, China) was maintained in DMEM with 10% FBS with antibiotics. Cultures were maintained at 37°C in a humidified atmosphere containing 5% CO_2_.

### Cell viability

Cell viability was determined by CCK-8 assay. EA.hy926 cells were grown in 96-well plates. CCK-8 solution (10 μl) was added to each well, followed by incubation for 4 h at 37°C. The absorbance at 450 nm was measured using a microplate reader (Multiskan Mk3, Thermo Labsystems, Chicago, IL, USA). Cell viability was expressed as a percentage of that of control (untreated) cells. Three independent experiments were performed in triplicate.

### Western blotting

Aortic tissues and EA.hy926 cells were retrieved and subjected to disruption using a homogenizer in lysis buffer containing 50 mM Tris-HCl, pH 7.5, 150 mM NaCl, 1% Nonidet P-40, 0.5% deoxycholic acid, 0.1% sodium dodecyl sulfate (SDS), 1 mM phenylmethylsulfonyl fluoride, and 100 μg/ml leupeptin and analyzed by SDS-polyacrylamide gel electrophoresis. Immunoblotting was carried out by incubation with rabbit polyclonal anti-VE-cadherin antibody (1:1000), rabbit polyclonal anti-phospho-Src (Tyr416) antibody (1:800) and rabbit polyclonal anti-Src antibody (1:1000), rabbit polyclonal anti-STIM1 antibody (1:1000), anti-human Orai1 antibody(1:1000), anti-GAPDH antibody (1:2000) and anti-Na,K-ATPase α1 antibody (1:2000), overnight at 4°C. HRP-conjugated secondary antibodies were used at 1: 5000 dilution for 1 h at 37°C and detected by enhanced chemiluminescence (Amersham Biosciences, UK).

### VE-cadherin immunofluorescence

Cells were grown on petri dishes and treated with HMGB1. Cells were then fixed in cold paraformaldehyde followed by extraction in 0.2% Triton X-100. Distribution of VE-cadherin was detected using rabbit anti-human VE-cadherin antibody and FITC-labeled goat anti-rabbit antibody as the secondary antibody. After subsequent washes, cells were incubated with DAPI at room temperature for 5 min. Cells were mounted in anti-photobleaching medium. Images were analyzed with a FV1000 confocal microscope system (Olympus, Tokyo, Japan). All pictures are representative of three independent experiments.

### Measurement of transendothelial electrical resistance

EA.hy926 cells were set up as for the endothelial permeability assay to measure transendothelial electric resistance (TER). EA.hy926 cell TER values ware obtained using a Millicell ERS-2 apparatus (Millipore, Bedford, MA, USA), following the manufacturer’s instructions. Coated inserts without cells were used as a blank. The electrical resistance of each insert with the appropriate treatment was calculated by subtracting the blank value from each reading. Each experiment was run in triplicate, and the resistance was measured twice for each well.

### Measurement of intracellular free Ca^2+^

Intracellular Ca^2+^ concentration was measured using the Ca^2+^-sensitive fluorescent indicator, Fluo-4/AM under an FV1000 confocal microscope. Briefly, EA.hy926 cells were incubated with 2 μM Fluo-4/AM at 37°C for 30 min in the dark in HBSS (pH 7.4) containing 5 mM KCl, 0.4 mM KH_2_PO_4_, 0.8 mM MgSO_4_, 137 mM NaCl, 0.3 mM Na_2_HPO_4_, 5.5 mM glucose, 1.26 mM CaCl_2_, 0.5 mM MgCl_2_. Ca^2+^-free buffer solution was prepared by omitting the CaCl_2_ and adding 0.3 mM EGTA. Fluo-4 was excited by an argon laser at 488 nm, and the emitted fluorescence was recorded through a 525 nm channel. Changes in intracellular Ca^2+^ (Δ[Ca^2+^]i) were estimated as ΔF/F_0_, where ΔF was defined as the fluorescence intensity after subtracting the basal intensity, and F0 was derived from the average intensity of the first 10–20 frames minus the background in the cell-free region.

### RNA interference

Two siRNAs against STIM1 (STIM1 siRNA-1: 5’-GGCUCUGGAUACAGUGCUCTT-3’ and STIM1 siRNA-2: 5’-GAAGCUGCGCGAUGAGAUCTT-3’) and Two siRNAs against Orai1 (Orai1 siRNA-1: 5’-CGAGCACTCCATGCAGGCG-3’ and Orai1 siRNA-2: 5’-GAAGCUGCGCGAUGAGAUCTT-3’) were designed and obtained from Shanghai GenePharma Co, Ltd. (Shanghai, China) and were transfected into EA.hy926 cells using a Lipo2000 Transfection Kit (Carlsbad, CA, USA). A scrambled, non-targeting siRNA (5’-UUCUCCGAACGUGUCACGUTT-3’ and 3’-ACGUGACACGUUCGGAGAATT-5’) was used as a control. Oligofectamine was diluted and mixed in OptiMEM I (Invitrogen) to allow the formation of siRNA-liposome complexes. After 20 min incubation at room temperature, the mixture was overlayed onto EA.hy926 cells cultured in medium without antibiotics. The transfected cells were analyzed 48 h after transfection.

### Statistical analysis

All data are shown as mean ± SD. The results were analyzed by one-way ANOVA followed by Fisher’s least significant difference for equal variances, and by Welch’s t-test followed by Dunnett’s T3 test for unequal variances. Statistical analysis was performed using SPSS 17.0 A value of P<0.05 was considered to be statistically significant.

## Results

### HMGB1 increased endothelial cell permeability

**Fig 1:**
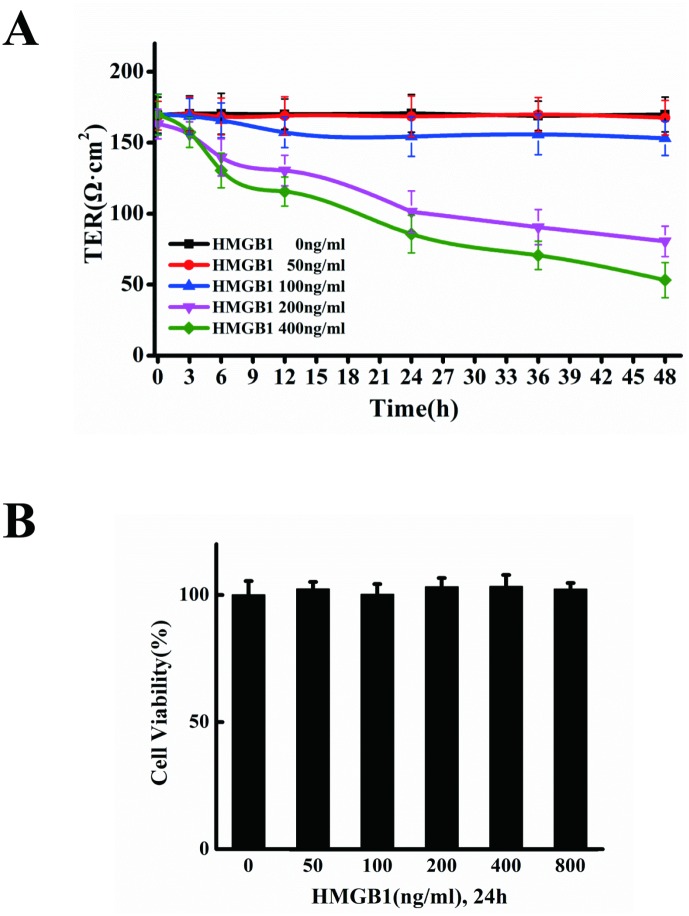
HMGB1 increases endothelial cell permeability. A. HMGB1-induced TEER decrease. EA.hy926 cells cultured on transwell filters were incubated for 3, 6, 12, 24, 36 and 48h, respectively, with or without 50, 100, 200 and 400 ng/ml HMGB1. The integrity of the tight junctions was assessed by measuring the TER. B. Cell viability in the cells treated by HMGB1. EA.hy926 cells were treated with 50, 100, 200, 400 and 800ng/ml HMGB1, respectively, for 24 h. The cell viability was measured by CCK-8 assay. Data are presented as mean ± SD of three independent experiments. *Indicates significant difference compared with the control group (P<0.05).

HMGB1 has been implicated in the progression of atherosclerosis [29]. HMGB1-treated vascular endothelial cells were used to investigate the mechanisms underlying atherosclerosis. We measured transendothelial electric resistance (TER) to clarify the influence of HMGB1 on permeability. 50 and 100ng/ml of HMGB1 has no effect, however, 200 ng/ml of HMGB1 caused a decrease in TER at 6 h of treatment and a significant drop at 12–48 h of treatment, in a time-dependent manner. 400 ng/ml of HMGB1 decreased TER starting from 6h (Fig 1A). Exposing the cells to 50–800 ng/ml of HMGB1 for 24h had no impact on cell viability, implying that the increase in permeability was not due to inhibition of cell viability (Fig 1B).

### HMGB1 disrupted VE-cadherin and intercellular gap formation

**Fig 2:**
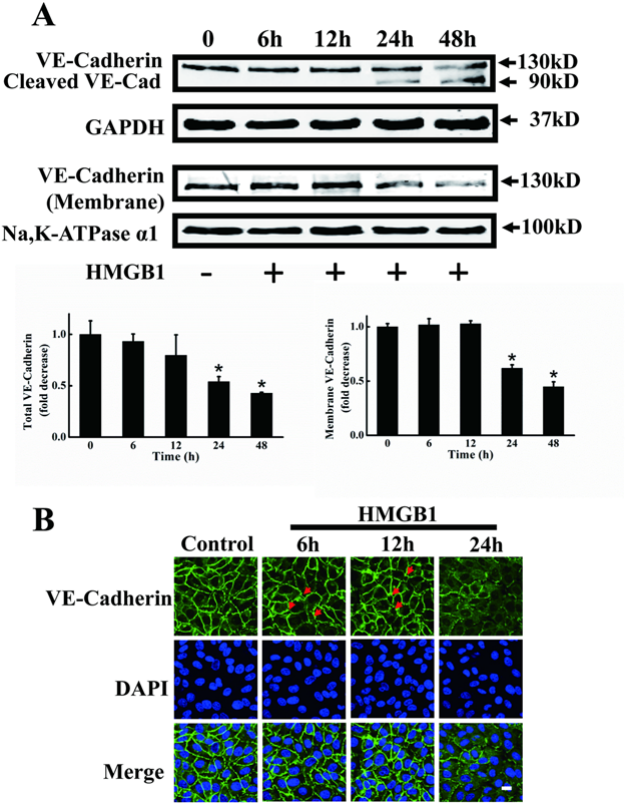
Disruption of VE-cadherin and intercellular gap formation in HMGB1-treated EA.hy926 cells. A. Representative immunoblots showing reduced expression of VE-cadherin protein by HMGB1. Total and cell membrane VE-cadherin protein levels were measured by western blotting in EA.hy926 cells treated with HMGB1 for 6, 12, 24 and 48 h, respectively. GAPDH and Na,K-ATPase α1 were used as loading controls for intact cells and plasma membranes, respectively. Western blots were quantified and analyzed statistically based on three independent experiments. *Indicates significant difference compared with wild-type group (P<0.05). B. HMGB1 increased intercellular gap formation. EA.hy926 cells were plated onto a Petri dish until the formation of a tight monolayer then treated with 200 ng/ml HMGB1 for 6, 12 and 24 h, respectively. The cells were fixed and distribution of VE-cadherin was detected using rabbit anti-human VE-cadherin antibody and FITC-labeled goat anti-rabbit antibody. Nuclei were stained with DAPI. Red arrows indicate intercellular gaps. A merged picture is shown for each condition. A representative field for each condition was captured using an Olympus FV1000 confocal microscope. Scale bar = 10 μm.

VE-cadherin is associated with atherosclerosis, and its dysfunction has a proatherogenic effect on vessels [30]. We investigated changes in VE-cadherin protein expression and morphology in HMGB1-treated vascular endothelial cells. VE-cadherin expression was unaffected at 6 and 12 h of treatment, but significant decreases in both total VE-cadherin and cleavage of VE-cadherin were observed after 24 h treatment of HMGB1 (Fig 2A). VE-cadherin plays a key role in endothelial barrier function at the plasma membrane. VE-cadherin expression at the plasma membrane was dramatically decreased by 24 and 48 h treatment with HMGB1 (Fig 2A). Immunofluorescence staining for VE-cadherin showed that VE-cadherin was mostly localized to the lateral cell surfaces in control endothelial cell monolayers, which contained continuous VE-cadherin and tight cell-to-cell contacts without intercellular gaps. Immunofluorescence staining for membrane-associated VE-cadherin showed no difference at 6 and 12 h of treatment, but decreased immunofluorescence was found in endothelial cells exposed to 200 ng/ml HMGB1 for 24 h. Intercellular gap formation was slightly disrupted starting from 6 h of treatment, and there was considerable morphologic diversity among adherens junctions at 24 h of treatment (Fig 2B).

### HMGB1 induced activation of Src

**Fig 3:**
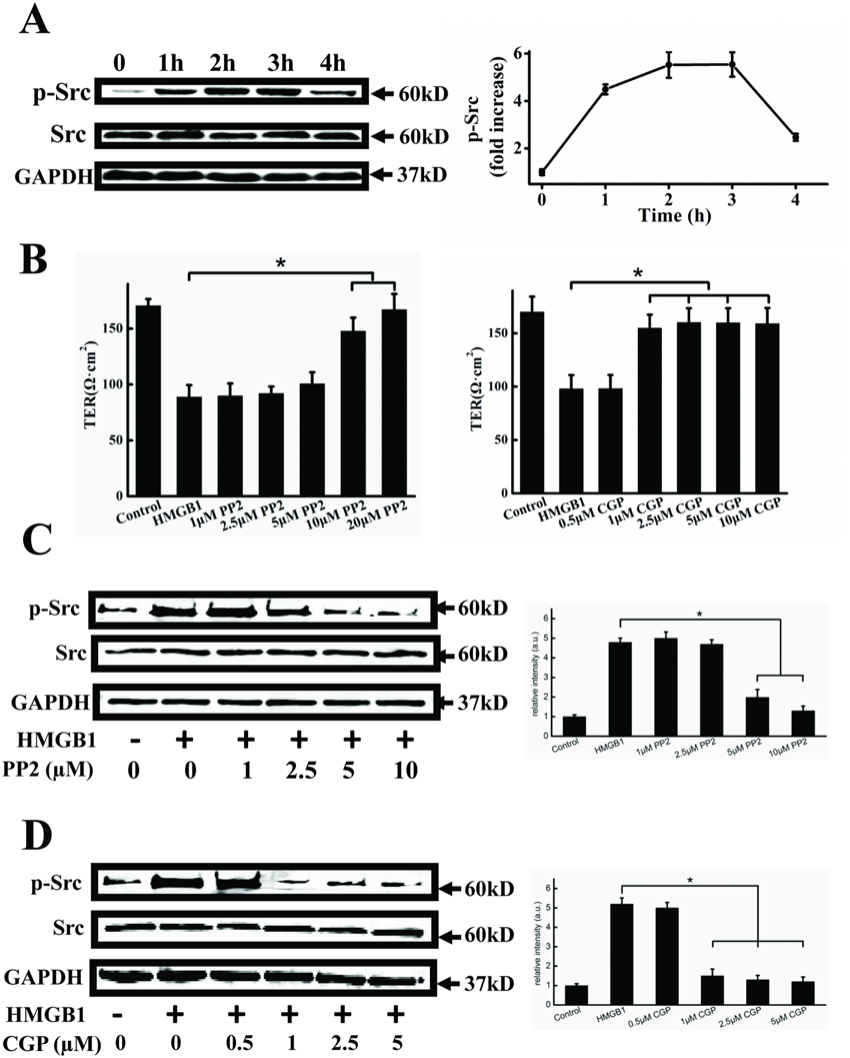
HMGB1 induces Src activation. A. Representative immunoblots showing HMGB1-induced Src activation. EA.hy926 cells were treated with 200 ng/ml HMGB1 for 1, 2, 3 and 4h, respectively. Cell lysates were analyzed by SDS-PAGE followed by western blotting using antibodies against phosphorylated Src and Src. B. PP2 and CGP77675 inhibit HMGB1-induced permeability. EA.hy926 cells were plated in the upper part of transwell chambers until the formation of a tight monolayer. The cells were preincubated with 1, 2.5, 5, 10 or 20μM PP2 (upper) or 0.5, 1, 2.5, 5 or 10 μM CGP77675 (lower) for 1 h, respectively. HMGB1 200 ng/ml was then added and the cells were incubated for an additional 24 h. After incubation, the integrity of the tight junctions was assessed by measuring the TER. Representative immunoblots showing that PP2 (C) and CGP77675 (D) decreased HMGB1-induced Src phosphorylation. Cells were preincubated with 1, 2.5, 5, or 10μM PP2 or 0.5, 1, 2.5 or 10 μM CGP77675 for 1 h, respectively. 200 ng/ml HMGB1 was then added and cells were incubated for an additional 24h. Cell lysates were analyzed by SDS-PAGE followed by western blotting using antibodies against phosphorylated Src and Src. GAPDH was used as a loading control. Western blots were quantified and analyzed statistically based on three independent experiments. Data are presented as mean ± SD of three independent experiments. *Indicates significant difference compared with the control group (P<0.05).

HMGB1-induced hyperpermeability has been reported to be mediated through the Src family of tyrosine kinase [13]. We therefore measured Src phosphorylation subsequent to HMGB1 treatment to determine if HMGB1 could activate this signaling pathway in EA.hy926 endothelial cells. Src was phosphorylated, with a peak at 2–3 h, followed by a decrease (Fig 3A). Moreover, we used the Src inhibitors PP2 and CGP77675 to investigate the role of Src in HMGB1-induced cell hyperpermeability. As shown in Fig 3B, both 10–20 μM PP2 and 1–10 μM CGP77675 significantly decreased HMGB1-induced cell hyperpermeability. Furtheremore, we detected whether the Src inhibitors PP2 and CGP77675 could inhibit the Src activation. Both 5–10 μM PP2 and 1–5 μM CGP77675 significantly blocked HMGB1-induced Src phosphorylation (Fig 3C and 3D).

### SOCE inhibitors inhibited HMGB1-induced Ca^2+^ influx, cell hyperpermeability and activation of Src

**Fig 4:**
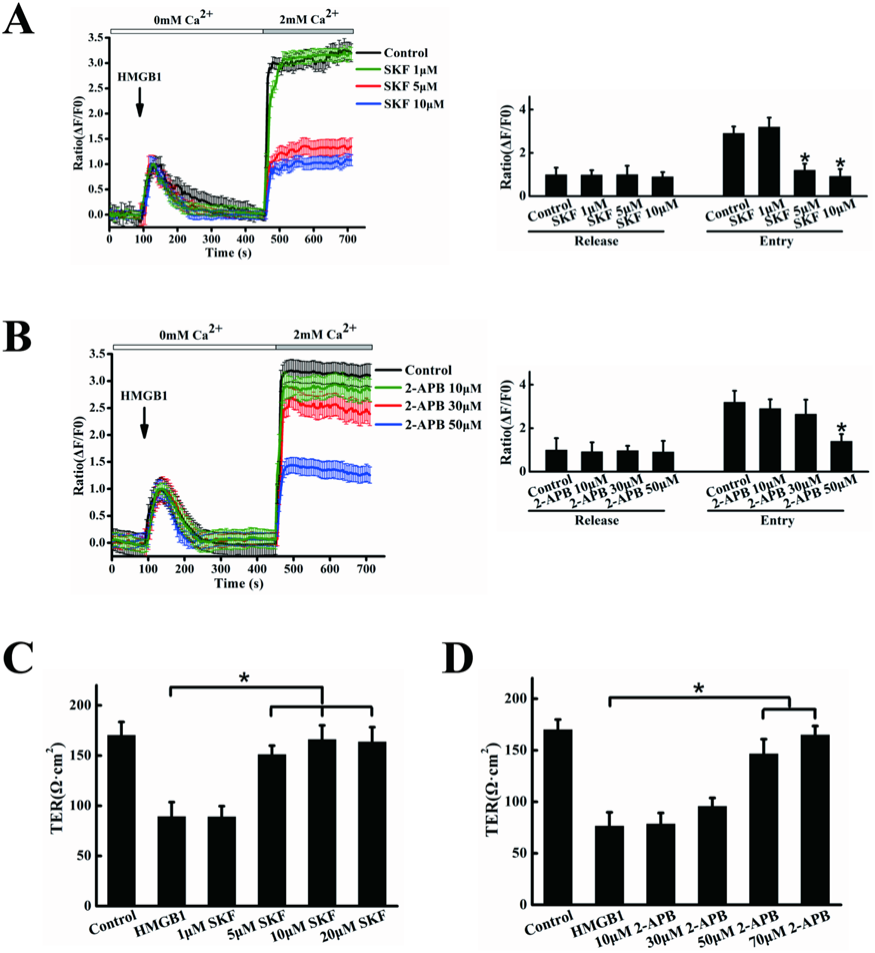
SKF96365 and 2-APB reduce Ca2+ influx and HMGB1-induced permeability. EA.hy926 cells were preincubated with 1, 5, 10 μM SKF96365 (A), or 10, 30, 50 μM 2-APB(B) or vehicle (DMSO), then stimulated with 200 ng/ml HMGB1, followed by the addition of 2 mM CaCl2. Intracellular calcium transients were measured using an Olympus FV1000 confocal microscope. Peak intracellular Ca2+ was quantified during intracellular release or extracellular Ca2+ influx. EA.hy926 cells were plated in the upper part of transwell chambers until the formation of a tight monolayer. The cells were preincubated with 1, 5, 10, 20 μM SKF96365 (C), or 10, 30, 50, 70 μM 2-APB (D) for 1 h, respectively. HMGB1 200 ng/ml was then added and the cells were incubated for an additional 24 h. After incubation, the integrity of the tight junctions was assessed by measuring the TER. Data are presented as mean ± SD of three independent experiments. *Indicates significant difference compared with the control group (P<0.05).

**Fig 5:**
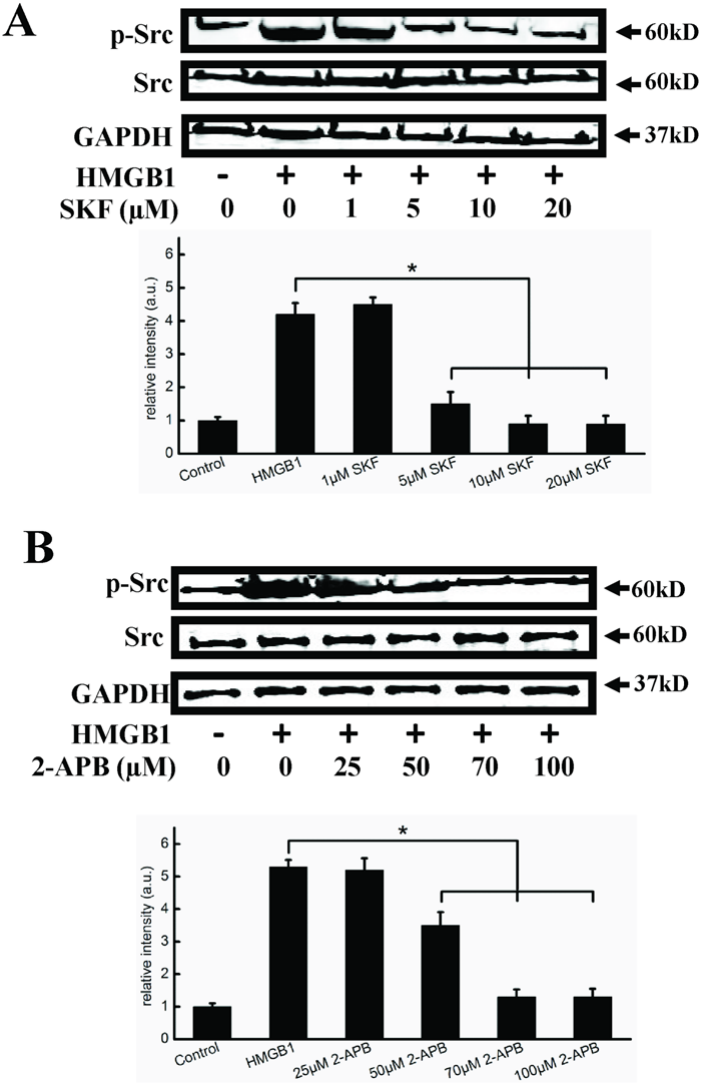
SKF96365 and 2-APB inhibit Src activation. Representative immunoblots showing that SKF96365 (A) and 2-APB (B) decreased HMGB1-induced Src phosphorylation. Cells were preincubated with 1, 5, 10, 20 μM SKF96365, or 25, 50, 70, 100 μM 2-APB for 1 h, respectively. 200 ng/ml HMGB1 was then added and cells were incubated for an additional 2 h. Cell lysates were analyzed by SDS-PAGE followed by western blotting using antibodies against phosphorylated Src and Src. GAPDH was used as a loading control. Western blots were quantified and analyzed statistically based on at least three independent experiments. Data are presented as mean ± SD of three independent experiments. *Indicates significant difference compared with the control group (P<0.05).

Evidence suggests that regulation of endothelial permeability is a complex process regulated by SOCE [31]. We used the SOCE inhibitors SKF96365 and 2-APB to investigate the role of SOCE in HMGB1-induced changes in cytosolic Ca^2+^. HMGB1 caused an initial increase in cytosolic Ca^2+^, with a rapid Ca^2+^ influx into cells following the addition of extracellular Ca^2+^. The apparent Ca^2+^ influx was significantly reduced in cells pretreated with 5–10 μM SKF96365 and 50 μM 2-APB, respectively, without affecting Ca^2+^ store release(Fig 4A and 4B). We further investigated the effects of SKF96365 and 2-APB on HMGB1-induced permeability. Endothelial cells were exposed to 1–20 μM SKF96365 or 10–70 μM 2-APB prior to HMGB1 treatment. As shown in Fig 4C and 4D, both SKF96365 and 2-APB significantly decreased HMGB1-induced cell hyperpermeability at 5–20 μM and 50–70 μM, respectively (P<0.05). Because SOCE inhibitors decreased HMGB1-induced endothelial cell hyperpermeability, in which Src was activated, we determined if SKF96365 and 2-APB might inhibit cell hyperpermeability by regulating the Src signaling pathway. As shown in Fig 5A and 5B, both SKF96365 and 2-APB significantly inhibited Src phosphorylation at 5–20 μM and 50–100 μM, respectively (P<0.05).

### Knockdown of STIM1 reduced HMGB1-induced Ca^2+^ influx, permeability and Src activation

**Fig 6:**
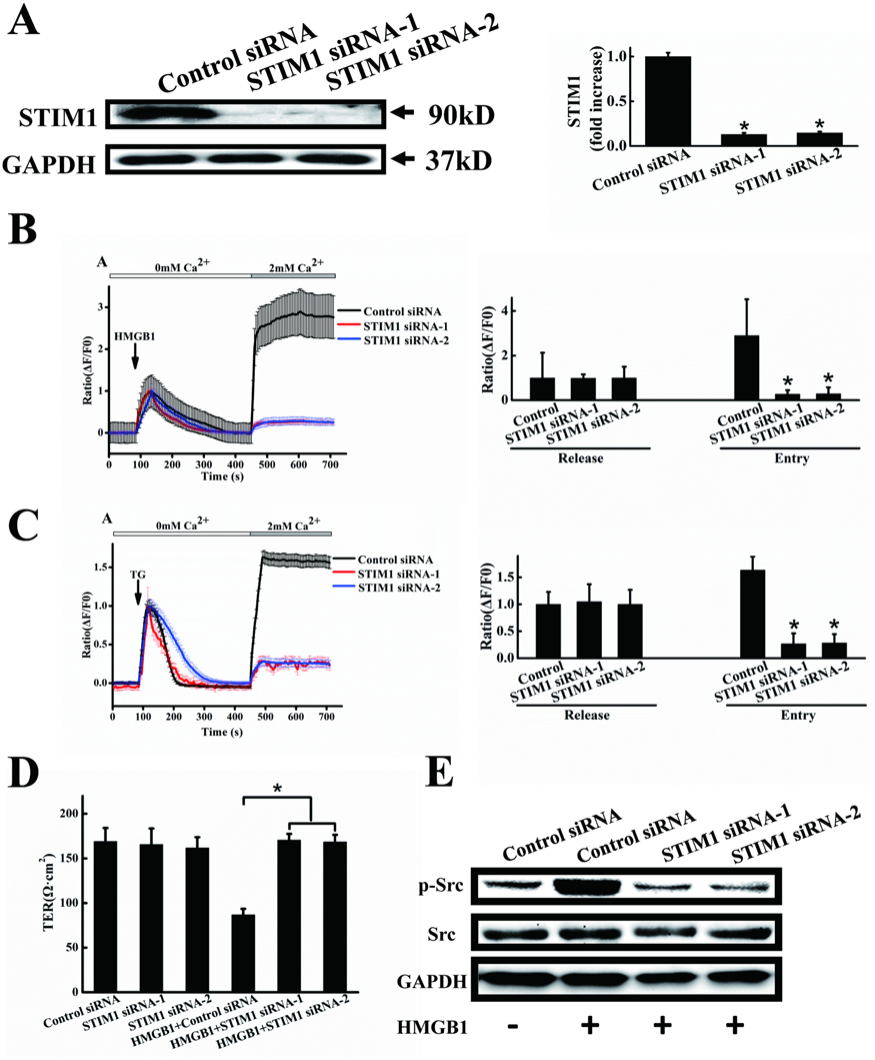
STIM1 knockdown decreases Ca2+ influx, HMGB1-induced permeability and Src phosphorylation. A. STIM1 protein expression after RNA inference. EA.hy926 cells were transfected for 48 h with STIM1 siRNA-1, siRNA-2 or control (scrambled) siRNA. Cells were harvested and total protein was extracted and subjected to western blotting with anti-STIM1 antibodies, with anti-GAPDH antibodies as a loading control. STIM1 expression was quantified and analyzed statistically based on three independent experiments. Transfected cells were also stimulated with 200 ng/ml HMGB1 (B) or 1 μM TG (C), followed by the addition of 2 mM CaCl2. Intracellular calcium transients were measured using an Olympus FV1000 confocal microscope. Peak intracellular Ca2+ was quantified during intracellular release or extracellular Ca2+ influx. D. HMGB1-induced permeability was inhibited by STIM1 knockdown. EA.hy926 cells were plated in the upper part of transwell chambers until the formation of a tight monolayer, then transfected with STIM1 siRNA-1, siRNA-2 or control (scrambled) siRNA. HMGB1 200 ng/ml was added and cells were incubated for an additional 24 h. After incubation, endothelial permeability was assessed, as described above. E. Representative immunoblots showing that STIM1 knockdown inhibits Src activation. Transfected cells were treated with or without 200 ng/ml HMGB1 for 2 h. Cell lysates were analyzed by SDS-PAGE followed by western blotting using antibodies against phosphorylated Src and Src. Data are presented as mean ± SD of three independent experiments. *Indicates significant difference compared with the control group (P<0.05).

Previous reports have shown that the conserved and ubiquitously-expressed protein STIM1 plays an essential role in store-operated calcium channel (SOC)-regulated influx, and may be a common component of SOCs [21]. We therefore used STIM1 siRNA to clarify its role in HMGB1-induced permeability. As shown in Fig 6A, transfection of EA.hy926 endothelial cells with two STIM1 siRNAs, but not with scrambled siRNA, significantly blocked the expression of STIM1 protein. STIM1 protein expression levels were reduced by approximately 90% in cells treated with the STIM1 siRNAs, compared with cells treated with scrambled siRNA. HMGB1-induced Ca^2+^ influx was significantly reduced by around 90% in cells treated with two STIM1 siRNAs, without affecting Ca^2+^ store release (Fig 6B). Thapsigargin (TG) is frequently used to induce SOCE by blocking the sarcoendoplasmic reticulum Ca^2+^-ATPases. We therefore determined the effects of knockdown of STIM1 on TG-induced SOCE. Ca^2+^ influx was significantly reduced by about 80% in cells treated with two STIM1 siRNAs, without affecting Ca^2+^ store release (Fig 6C). We further investigated the effects of STIM1 siRNA on HMGB1-induced permeability and Src activation. siRNA knockdown of STIM1 had no effect on endothelial cell permeability, but HMGB1-induced endothelial cell hyperpermeability was significantly suppressed (Fig 6D). Src activation induced by HMGB1 was also significantly inhibited by knockdown of STIM1 (Fig 6E).

#### Orai1 knockdown inhibited HMGB1-induced permeability

**Fig 7:**
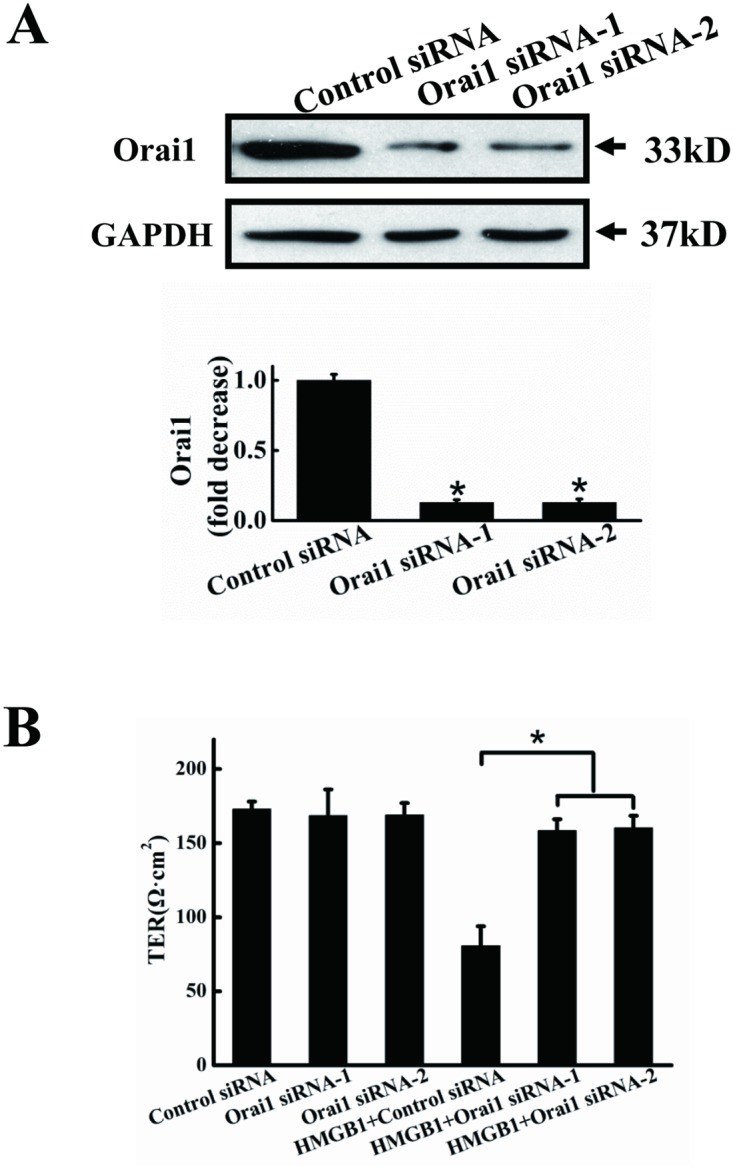
Orai1 knockdown decreases HMGB1-induced permeability. A. Orai1 protein expression after RNA inference. EA.hy926 cells were transfected for 48 h with Orai1 siRNA-1, Orai1 siRNA-2 or control (scrambled) siRNA. Cells were harvested and total protein was extracted and subjected to western blotting with anti-Orai1 antibodies, with anti-GAPDH antibodies as a loading control. Orai1 expression was quantified and analyzed statistically based on three independent experiments. B. HMGB1-induced permeability was inhibited by Orai1 knockdown. EA.hy926 cells were plated in the upper part of transwell chambers until the formation of a tight monolayer, then transfected with Orai1 siRNA-1, Orai1 siRNA-2 or control (scrambled) siRNA. HMGB1 200 ng/ml was added and cells were incubated for an additional 24 h. After incubation, endothelial permeability was assessed, as described above. Data are presented as mean ± SD of three independent experiments. *Indicates significant difference compared with the control group (P<0.05).

Since STIM1 may promote endothelial permeability independently of Ca2+ entry[32], Orai1, one of key molecules in SOCE[33], was downregulated by siRNA to confirm crucial role of calcium entry in the HMGB1-induced cell hyperpermeability. HMGB1-induced endothelial cell hyperpermeability was significantly inhibited by knockdown of Orai1 (Fig 7). As shown in Fig 7A, transfection of EA.hy926 endothelial cells with two Orai1 siRNAs, but not with scrambled siRNA, significantly blocked the expression of Orai1 protein. Orai1 protein expression levels were reduced by approximately 85% in cells treated with the Orai1 siRNAs, compared with cells treated with scrambled siRNA. We further investigated the effects of Orai1 siRNA on HMGB1-induced permeability. siRNA knockdown of Orai1 had no effect on endothelial cell permeability, but HMGB1-induced endothelial cell hyperpermeability was significantly suppressed (Fig 7B).

## Discussion

The present study indicates the existence of a novel mechanism responsible for modulating vascular permeability. Our results demonstrated the following: (1) HMGB1 increased endothelial cell permeability in time- and concentration- dependent manners; (2) VE-cadherin protein expression was decreased and detectable intercellular gaps were induced in endothelial cells treated with HMGB1; (3) Inhibition of SOCE or STIM1 knockdown significantly attenuated HMGB1-induced Ca^2+^ influx, endothelial cell hyperpermeability and Src activation,. Taken together, these data suggest that SOCE, acting through STIM1, plays an important role in the regulation of HMGB1-stimulated endothelial cell hyperpermeability through Src activation.

HMGB-1 is a chromatin-associated nuclear protein that stabilizes nucleosome formation and facilitates transcription-factor binding by bending DNA [34]. HMGB1 has been described as an early proinflammatory factor and plays a crucial role in many pathophysiological processes, such as sepsis, rheumatoid arthritis and liver injury [13, 35]. HMGB1 expression was recently reported to be markedly increased in areas adjacent to the necrotic core of atherosclerotic lesions, and to potentially be released from several cell types in the atherosclerotic plaque, including smooth muscle cells, endothelial cells, macrophages and activated platelets[36]. The importance of HMGB1 in the development of atherosclerosis has been demonstrated in apoE^-/-^ transgenic mice[37]. In the current study, HMGB1 increased endothelial hyperpermeability, which was consistent with recent data suggesting that HMGB1 is involved in the pathophysiology of hyperpermeability of endothelial cell monolayers in sepsis[13]. VE-cadherin is the major determinant of endothelial cell contact integrity and plays a vital role in the control of vascular permeability in endothelial cells [38]. Previous studies showed that VE-cadherin was mediated by inflammatory factors during cytokine-induced permeability and endothelial cell gap formation [39]. The expression of membrane VE-cadherin was decreased in HUVECs treated by HMGB1 at 12h and 24h, and restored at 48h of treatment. However, the total VE-cadherin had no change [13]. Our data showed that both total and cell surface expression of VE-cadherin were decreased in endothelial cells after 24 h treatment of HMGB1. However, intercellular gap formation was slightly disrupted starting from 6 h of treatment, and there was considerable morphologic diversity among adherens junctions at 24 h of treatment These data imply that the disruption of intercellular gap formation is not associated with the loss of VE-cadherin from the cell membrane and the loss of membrane VE-cadherin appeared to be at least partly the result of loss of VE-cadherin protein, which is inconsistent with previous reports[13]. Further studies are needed to address this issue.

Endothelial permeability is a complex process that is regulated by SOC in chronic pulmonary hypertension [31]. The rise in intracellular Ca^2+^ through transient receptor potential channel 1, the essential component of SOC, has been established as the initial pivotal signal that precedes endothelial cell cytoskeletal reorganization and the disassembly of VE-cadherin [18, 40]. In the present study, HMGB1-induced endothelial hyperpermeability was attenuated by the pharmacological inhibitors SKF96365 and 2-APB, suggesting that a signal channel is required to regulate endothelial permeability. SKF96365 and 2-APB also inhibited TG-induced SOCE (data not shown) and HMGB1-induced extracellular Ca^2+^ influx in endothelial cells, without affecting intracellular Ca^2+^ store release. These results reveal that SKF96365 and 2-APB reduced endothelial hyperpermeability by inhibiting Ca^2+^ influx via SOCE. However, although SKF96365 and 2-APB have been widely used to inhibit SOCE, they are both non-specific and can also suppress voltage-operated and receptor-operated Ca^2+^ channels [41, 42]. STIM1 is a sensor of Ca^2+^ concentration in the lumen of the ER, which plays a vital role in mediating SOCE [43, 44]. When a cell is at rest, Ca^2+^-bound STIM1 molecules are located predominantly in the ER but also appear in the plasma membrane. Upon Ca^2+^ depletion of the ER as a result of cell activation, STIM1 in the ER becomes aggregated into puncta, relocated in distinct regions of the ER close to the plasma membrane, and associated and reorganized in the plasma membrane, which is sufficient to activate SOCs and mediate Ca^2+^ influx. Recent studies indicate that STIM1 is required for endothelial SOCE [45, 46]. In the present study, we confirmed that knockdown of STIM1 by siRNA inhibited TG-induced SOCE in endothelial cells. Furthermore, STIM1 knockdown significantly reduced HMGB1-induced endothelial hyperpermeability as well as extracellular Ca^2+^ influx without affecting intracellular Ca^2+^ store release. In addition, STIM1 was recently reported to promote endothelial permeability independently of Ca^2+^ entry [32]. Orai1 calcium channel, the critical molecule in SOCE [33, 47], was downregulated by siRNA to confirm crucial role of calcium entry in the HMGB1-induced cell hyperpermeability. Orai1 knockdown significantly inhibited HMGB1-induced endothelial hyperpermeability. This provides further evidence to support the involvement of SOCE in endothelial hyperpermeability and Ca^2+^ influx.

Recent evidence has shown that Src family of kinases is associated with the regulation of microvascular barrier function and various endothelial responses, including hyperpermeability, to different proinflammatory mediators [48, 49]. Other authors have shown that HMGB1-induced hyperpermeability was mediated through the RAGE receptor and Src family tyrosine kinase pathway [13]. However, in the present study, one of the Src inhibitor, PP2, significantly decreased HMGB1-induced cell hyperpermeability at a concentration of 10μM, while 5μM PP2 could inhibit Src activity, which implied that non-specific inhibition probably prevents HMGB1-induced loss of TER independently of Src. Moreover, SKF96365, 2-APB and STIM1 knockdown were all able to inhibit HMGB1-induced phosphorylation of Src, suggesting that HMGB1-stimulated activation of Src may depend on SOCE.

The results of this study indicate the existence of a novel mechanism for modulating vascular permeability: HMGB1 activates Ca^2+^ entry via SOCE, which in turn activates the Src pathways. In addition to indicating the relevance of SOCE to endothelial permeability, this study provides the first evidence for STIM1 involvement in the signaling pathways regulating HMGB1-induced endothelial hyperpermeability.
